# Dapagliflozin treatment is associated with a reduction of epicardial adipose tissue thickness and epicardial glucose uptake in human type 2 diabetes

**DOI:** 10.1186/s12933-023-02091-0

**Published:** 2023-12-19

**Authors:** Francesca Cinti, Lucia Leccisotti, Gian Pio Sorice, Umberto Capece, Domenico D’Amario, Margherita Lorusso, Shawn Gugliandolo, Cassandra Morciano, Andrea Guarneri, Maria Angela Guzzardi, Teresa Mezza, Amedeo Capotosti, Luca Indovina, Pietro Manuel Ferraro, Patricia Iozzo, Filippo Crea, Alessandro Giordano, Andrea Giaccari

**Affiliations:** 1 Centro Malattie Endocrine e Metaboliche, Dipartimento di Scienze Mediche e Chirurgiche, Fondazione Policlinico Universitario A. Gemelli IRCCS and Università Cattolica del Sacro Cuore, Rome, Italy; 2 UOC di Medicina Nucleare, Dipartimento di Diagnostica per Immagini, Radioterapia Oncologica ed Ematologia, Fondazione Policlinico Universitario A. Gemelli IRCCS and Università Cattolica del Sacro Cuore, Rome, Italy; 3 Sezione di Medicina Interna, Endocrinologia, Andrologia e Malattie Metaboliche, Dipartimento di Medicina di Precisione e Rigenerativa e Area Jonica - (DiMePRe-J), Università Degli Studi di Bari “Aldo Moro”, Bari, Italy; 4 Dipartimento di Scienze Cardiovascolari, UOC Di Cardiologia, Fondazione Policlinico Universitario A. Gemelli IRCCS, and Università Cattolica del Sacro Cuore, Rome, Italy; 5 Università del Piemonte Orientale , Dipartimento Medicina Translazionale, Novara, Italy; 6 Dipartimento di Medicina e Chirurgia Traslazionale, Università Cattolica del Sacro Cuore, Rome, Italy; 7 Dipartimento di Scienze Cliniche e Sperimentali, Medicina Interna - Università degli Studi di Brescia, Brescia, BS Italy; 8 Istituto di Fisiologia Clinica, Consiglio Nazionale delle Ricerche (CNR), Pisa, Italy; 9 Pancreas Unit, CEMAD Centro Malattie dell’Apparato Digerente, Medicina Interna e Gastroenterologia, Fondazione Policlinico Universitario Gemelli IRCCS, Rome, Italy; 10 UOSD Fisica Medica e Radioprotezione, Dipartimento di Diagnostica per Immagini, Fondazione Policlinico Universitario A. Gemelli IRCCS, Radioterapia Oncologica ed Ematologia, Rome, Italy; 11 U.O.S. Terapia Conservativa della Malattia Renale Cronica, Fondazione Policlinico Universitario A. Gemelli IRCCS, Università Cattolica del Sacro Cuore, Rome, Italy

**Keywords:** Diabetes, Metabolism, Epicardial adipose tissue, PET, SGLT-2i, Microvascular dysfunction, Precision medicine

## Abstract

**Objective:**

We recently demonstrated that treatment with sodium-glucose cotransporter-2 inhibitors (SGLT-2i) leads to an increase in myocardial flow reserve in patients with type 2 diabetes (T2D) with stable coronary artery disease (CAD). The mechanism by which this occurs is, however, unclear. One of the risk factors for cardiovascular disease is inflammation of epicardial adipose tissue (EAT). Since the latter is often increased in type 2 diabetes patients, it could play a role in coronary microvascular dysfunction. It is also well known that SGLT-2i modify adipose tissue metabolism. We aimed to investigate the effects of the SGLT-2i dapagliflozin on metabolism and visceral and subcutaneous adipose tissue thickness in T2D patients with stable coronary artery disease and to verify whether these changes could explain observed changes in myocardial flow.

**Methods:**

We performed a single-center, prospective, randomized, double-blind, controlled clinical trial with 14 T2D patients randomized 1:1 to SGLT-2i dapagliflozin (10 mg daily) or placebo. The thickness of visceral (epicardial, mediastinal, perirenal) and subcutaneous adipose tissue and glucose uptake were assessed at baseline and 4 weeks after treatment initiation by 2-deoxy-2-[^18^F]fluoro-D-glucose Positron Emission Tomography/Computed Tomography during hyperinsulinemic euglycemic clamp.

**Results:**

The two groups were well-matched for baseline characteristics (age, diabetes duration, HbA1c, BMI, renal and heart function). Dapagliflozin treatment significantly reduced EAT thickness by 19% (p = 0.03). There was a significant 21.6% reduction in EAT glucose uptake during euglycemic hyperinsulinemic clamp in the dapagliflozin group compared with the placebo group (p = 0.014). There were no significant effects on adipose tissue thickness/metabolism in the other depots explored.

**Conclusions:**

SGLT-2 inhibition selectively reduces EAT thickness and EAT glucose uptake in T2D patients, suggesting a reduction of EAT inflammation. This could explain the observed increase in myocardial flow reserve, providing new insights into SGLT-2i cardiovascular benefits.

**Supplementary Information:**

The online version contains supplementary material available at 10.1186/s12933-023-02091-0.

## Introduction

Currently, sodium-glucose cotransporter-2 inhibitors (SGLT-2i) are one of the main treatments for reducing high cardiovascular (CV) mortality in patients with type 2 diabetes (T2D) [1]. However, the mechanism by which they provide CV protection remains unclear. We recently demonstrated that treatment with the SGLT-2i dapagliflozin in T2D patients with stable coronary artery disease (CAD), can increase myocardial flow reserve (MFR), and we speculated that this increase could be caused by an improvement in coronary microvascular dysfunction [2, 3]. Indeed, coronary microvascular dysfunction, in the absence of obstructive CAD and myocardial diseases, is considered a reversible condition, and MFR can be used to assess efficacy of treatment [4].

In a recent study, Mahmoud et al. suggested that epicardial adipose tissue (EAT) has a pivotal role in the pathogenesis of microvascular dysfunction [5]. EAT is a particular adipose tissue located in the atrioventricular and interventricular grooves around the coronary arteries (peri-coronary EAT) and over the myocardium (myocardial EAT) [6]. It has two fundamental anatomical and physiological characteristics: first, it is a visceral fat depot in direct contact with the myocardium (i.e., no muscle fascia separates it from the myocardium). Thus, the two tissues share the same microcirculation allowing a direct cross-talk [7]. Second, it presents features of both white and brown fat, which suggests that it is a metabolically active fat depot [8].

Indeed, EAT is considered a modifiable CV risk factor involved in the pathogenesis of CAD, atrial fibrillation, heart failure (HF) and has been recently recognized as a source of therapeutic targets and clinical biomarkers [9, 10]. Limited evidence suggests that SGLT-2i treatment can reduce EAT volume [11]. However, the links between these phenomena still need to be clarified.

Thus, the aim of this study was to evaluate whether treatment with the SGLT-2i dapagliflozin, compared to placebo, has an impact on adipose tissue in patients with T2D and stable CAD. To achieve this, we measured morphological (adipose tissue thickness) and metabolic (adipose tissue glucose uptake) changes and determined whether the latter were associated with the increase in MFR previously observed after a 4-week treatment with dapagliflozin [2].

We conducted a phase III multidisciplinary clinical study on a well-selected population of T2D patients with stable CAD (coronary stenosis ≥ 30% and < 80%), with or without previous percutaneous coronary intervention (> 6 months) but no HF, using 2-deoxy-2-[18F]fluoro-D-glucose (FDG) Positron Emission Tomography/Computed Tomography (PET/CT) to assess adipose tissue morphology and metabolism during a euglycemic hyperinsulinemic clamp.

## Research design and methods

Details of the study design of the DAPAHEART trial can be found in the protocol paper [12]. Here, we analyze the secondary outcome of the trial to evaluate changes from baseline in the thickness of visceral (pericardial, perirenal, mediastinal) and subcutaneous adipose tissue, expressed in centimeters, and to detect FDG uptake in visceral (pericardial, perirenal, mediastinal) and subcutaneous adipose tissue through whole body FDG PET/CT performed during hyperinsulinemic euglycemic clamp, expressed as maximum Standard Uptake Value (SUVmax) (changes from baseline).

The study was approved by the local ethics committee (Fondazione Policlinico Universitario Agostino Gemelli IRCCS, study protocol code GIA-DAP-16-005) and registered at eudract.ema.europa.eu (EudraCT No. 2016-003614-27) and ClinicalTrials.gov (NCT 03313752). Informed, written consent was obtained from all participants.

### Trial design and participants

This was a phase III, single-center, randomized, two-arm, parallel-group, double-blind, placebo-controlled study, consisting of a screening phase (days -14 to -1) and a 4-week double-blind, placebo-controlled treatment phase. The details of the trial design can be found in the previously published protocol paper [12]. Briefly, inclusion criteria were (1) T2D, (2) no previous history of myocardial infarction, (3) stable coronary artery disease (coronary stenosis ≥ 30% and < 80% in at least one native major coronary artery), with or without previous percutaneous coronary intervention (> 6 months), with no evidence of critical restenosis and no indication to myocardial revascularization according to the current guidelines of the European Society of Cardiology [13], (4) glycated hemoglobin [HbA1c]: 7–8.5% or 53–69 mmol/mol on stable standard of care anti-hyperglycemic regimen, (5) body mass index (BMI): 25 – 35 kg/m [2]. Exclusion criteria were: (1) type 1 diabetes or previous diagnosis of Latent Autoimmune Diabetes of Adults, (2) NYHA class III or IV, (3) reduced LVEF (≤ 50%), (4) unstable angina, (5) moderate to severe renal impairment (estimated glomerular filtration rate < 60 mL/min /1.73 m^2^) (6) coronary artery disease with a coronary stenosis ≥ 80% in a major coronary artery defined by invasive coronary angiography.

The study design is illustrated in Additional file 1: Fig. 1S.

**Fig. 1:**
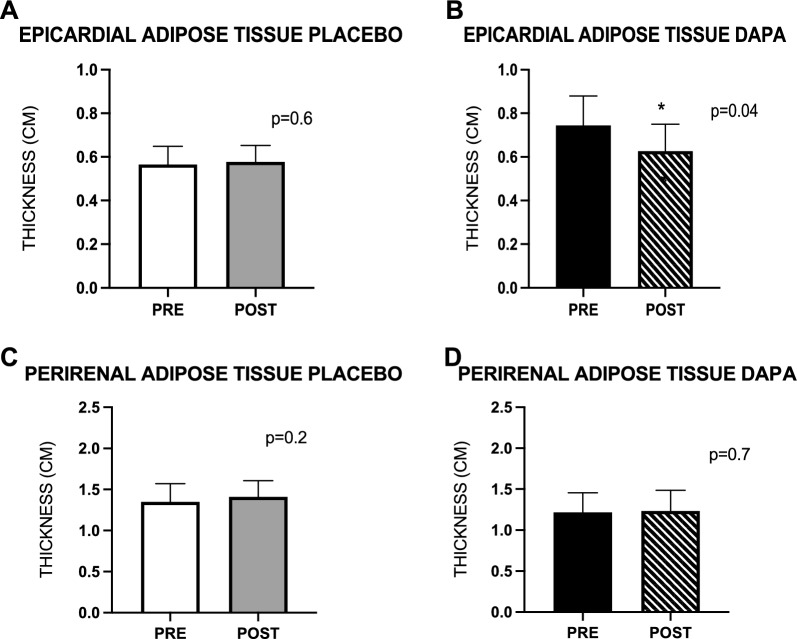
Epicardial and perirenal adipose tissue thickness (cm): placebo group (A–C) and dapagliflozin group (**B**–**D**). Data are mean ± SEM. * = p < 0.05

All participants were screened for all applicable inclusion and exclusion criteria, underwent baseline assessments, and were enrolled during the screening phase. Participants were randomly assigned in a 1:1 ratio to receive dapagliflozin (10 mg, orally, once daily) or placebo for 4 weeks. Randomization and packaging of medicine were handled by the hospital pharmacy. All baseline assessments were repeated at the end of treatment (4 weeks).

### PET imaging and analysis

PET/CT was performed at baseline and after 4 weeks of treatment using a 3D PET/CT scanner (Biograph mCT, Siemens Healthcare).

### FDG PET/CT during Euglycemic hyperinsulinemic clamp

The details of the imaging protocol of the DAPAHEART trial can be found in the protocol paper [12]. Briefly, all PET/CT studies were conducted after an overnight fast using a Biograph mCT (Siemens Healthcare) scanner. Prior to the PET/CT scan, patients were asked to void their bladder, and a polyethylene cannula was inserted in a superficial forearm vein for infusion of glucose, insulin and FDG. A second cannula was threaded into a superficial vein of the opposite arm or hand, for blood glucose sampling. At time 0, a primed-constant insulin infusion (40 mU·min^−1^·m^2^ of body surface area) was started. The prime consists of four times the final constant rate for the first 4 min, followed by two times the constant rate for 3 min. Euglycemia was maintained using a 20% glucose infusion adjusted according to frequent plasma glucose measurements. Blood samples for measuring chemistry and hormones were drawn at −10 min and at steady state. After at least 60 min of hyperinsulinemic-euglycemia, a 50-min dynamic PET/CT scan of the cardiac region was carried out after the i.v administration of FDG (185 MBq) over 15 s for myocardial glucose uptake (MGU) measurement. Finally, a CT transmission scan (50mAs, 120 kV, slice thickness of 3 mm, 2.80-slice increment) was acquired from the vertex to the mid-thigh for photon attenuation correction and anatomical localization. Whole body PET imaging was acquired in three-dimensional mode, 2 min per bed position, with a 256 × 256 matrix and pixel size/slice thickness of 3.18 × 3.18/5.00 mm. After normalization and correction for dead time, randoms, scatters and attenuation, PET data were reconstructed using an iterative algorithm (ordered-subsets expectation maximization, 2 iterations and 21 subsets), with the combined effect of point spread function (PSF) modelling and time of flight (TOF).

Whole body FDG PET/CT image analysis was performed using the PMOD software (PMOD Technologies LLC). Volumes of interest (VOIs, 0.16 cm^3^) were manually drawn in the fused PET/CT images using all three transaxial, sagittal and coronal planes to identify pericardial, perirenal and subcutaneous fat. Maximum standardized uptake value (SUVmax) and SUVmean were obtained in fat surrounding anterior interventricular artery (AIV), right coronary artery (RCA), left circumflex coronary (CX) and the roof of left atrium (RLA) as well as thoracic and pelvic subcutaneous fat (SCF), visceral thoracic fat (VTF) and perirenal fat (PRF) in dapagliflozin and placebo groups. Epicardial, mediastinal, subcutaneous and perirenal fat thickness were also measured (cm) on CT images. In particular, on unenhanced CT images, EAT thickness (cm) measurements were performed along the right ventricular anterior free wall in a single sagittal slice having the greatest EAT thickness [14]. Measurements were taken by two independent nuclear medicine physicians blind to the results with an interobserver variability of less than 5%.

## Statistical analysis

The details of sample size estimation and statistical analysis are available in the study design protocol paper [12]. Data are presented as mean ± SEM or median (95% Confidence Interval, CI) as appropriate. Data were examined for normal distribution. Parametric and/or non-parametric tests were used, as appropriate. Within group differences were assessed using the t-test (or equivalent non-parametric test) for paired data; between group differences were assessed using the t-test (or equivalent non-parametric test) for unpaired data. In addition, tests for repeated measurements were used to account for treatment versus group effects and interactions**.**

## Results

### Study population

The study screened 48 volunteers for eligibility and 16 participants were included and randomized to receive dapagliflozin (n = 8) or placebo (n = 8) (Additional file 1: Figure S2).

In two patients, all FDG data were excluded from the analysis because post-treatment FDG PET/CT images were not evaluable.

Demographic and baseline characteristics of each treatment group are summarized in Table 1.

**Table 1:** Baseline characteristics

	Placebo (n = 7)	Dapagliflozin 10 mg (n = 7)	P value
Male sex, N (%)	4 (57.1)	7 (100)	
Age, yeras	67 ± 2.4	65.1 ± 2.7	P = 0.6
Diabetes duration, years	8 ± 0.7	5.8 ± 0.9	P = 0.1
HbA1c, %	8 ± 0.2	7.8 ± 0.2	P = 0.5
Fasting glycemia (mg/dl)	121 ± 10.5	141.7 ± 11.3	P = 0.1
C-peptide (ng/ml)	1.2 ± 0.3	1.9 ± 0.2	P = 0.1
BMI, kg/ml^2^	29.1 ± 1.2	27.8 ± 1.1	P = 0.8
Body weight, kg	79.2 ± 4.3	83 ± 2.5	P = 0.4
Heart rate, bpm	70.4 ± 4.3	60.7 ± 4.4	P = 0.2
Systolic BP, mmHg	135.7 ± 5.6	145 ± 5.5	P = 0.35
Diastolic BP, mmHg	70 ± 3.9	69.2 ± 2.7	P = 0.9
CAD (previous PCI/no PCI)	4/3	2/5	P = 0.2
eGFR (ml/min)	93 ± 2.2	86.8 ± 6.7	P = 0.45
Medications			
Metformin	7 (100)	6 (85.7)	
DPP-4i	3 (42.2)	2 (28.5)	
GLP-1 RA	2 (28.5)	1 (14.2)	
Basal insulin	1 (14.2)	2 (28.5)	
Sulfonylurea	1 (14.2)	1 (14.2)	

Data are mean ± SEM. HbA1c, glycated hemoglobin; BMI, body mass index; CAD, coronary artery disease; eGFR, estimated glomerular filtration rate

Patient compliance was high, and no study drug-related severe adverse events were registered.

### Effect of dapagliflozin treatment on body weight and adipose tissue thickness

Compared with baseline, we did not observe any significant change in body weight between placebo and treatment group (79.28 ± 4.3 vs 81 ± 4.88 kg, p = 0.19 in placebo group; 83.14 ± 2.5 vs 82.55 ± 3,1 kg, p = 0.2 in dapagliflozin group).

We observed a significant 19% reduction of the thickness of EAT in the dapagliflozin group compared to the placebo group (0.74 ± 0.12 vs 0.60 ± 0.10 cm, p = 0.04 in dapagliflozin group; 0.56 ± 0.06 vs 0.55 ± 0.06 cm, p = 0.6) Fig. 1 A-B. The reduction in EAT thickness had no effect on myocardial insulin sensitivity (MGU), which was not statistically different from baseline in both the dapagliflozin and placebo group (2.22 ± 0.59 vs 1.92 ± 0.42 μmol/100 g/min, p = 0.41 compared with the placebo group 2.00 ± 0.55 vs 1.60 ± 0.45 μmol/100 g/min, p = 0.5) as previously reported [2]. The thickness of perirenal adipose tissue remained unchanged from baseline both in the dapagliflozin (1.21 ± 0.2 vs 1.17 ± 0.2, p = 0.7) and the placebo group (1.34 ± 0.2 vs 1.40 ± 0.18, p = 0.2) Fig. 1 C-D. The thickness of the other adipose tissue depots explored (mediastinal and subcutaneous) also remained unchanged (data not shown).

### Dapagliflozin treatment reduced epicardial adipose tissue FDG SUV

**Fig. 2:**
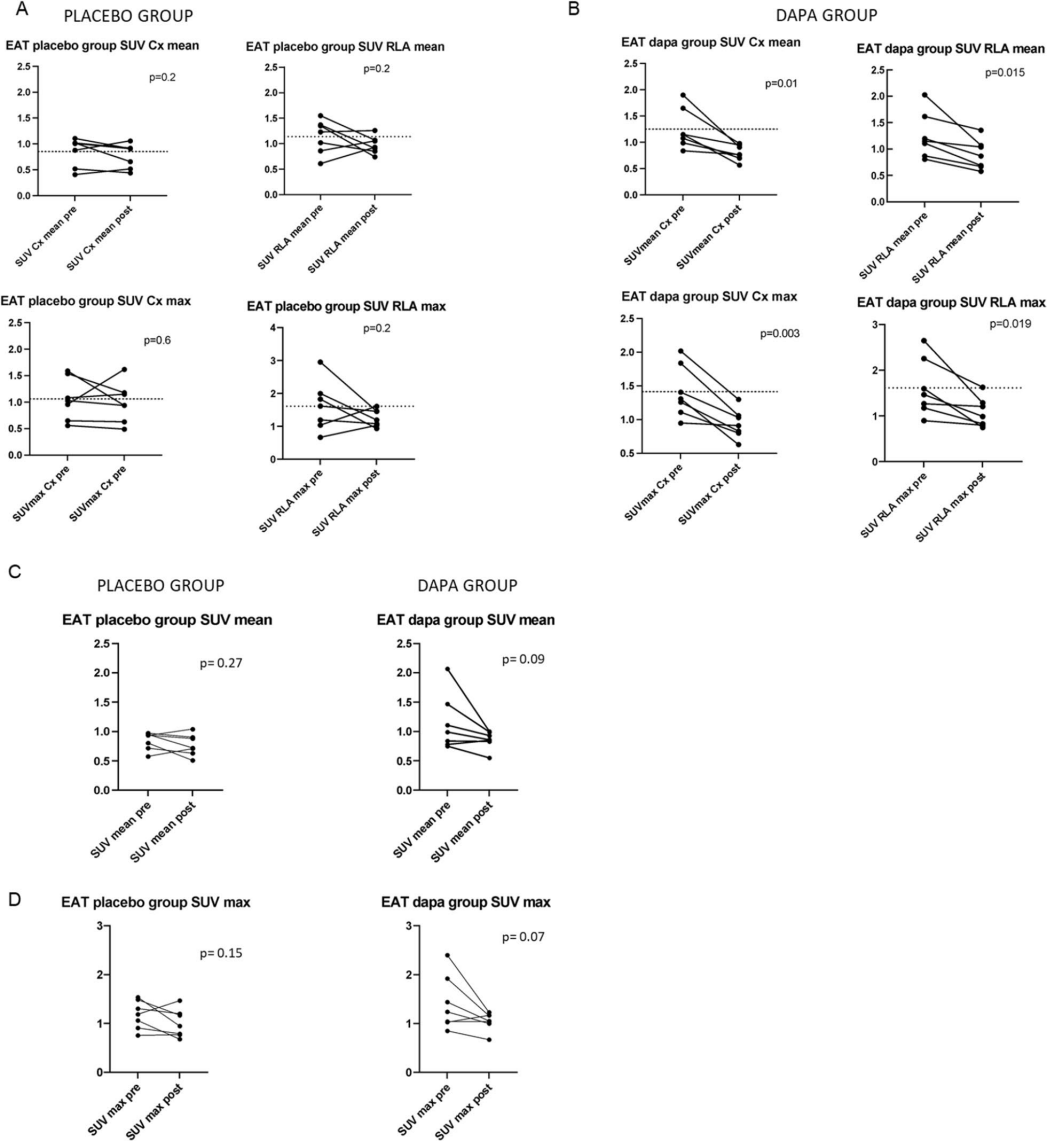
Epicardial adipose tissue (EAT) metabolism: SUV max and SUV mean at left circumflex artery level (Cx) and at the roof of the left atrium (RLA) in placebo (**A**) and dapagliflozin group (**B**); SUV mean (**C**) and SUV max (**D**) of EAT at right coronary artery/left circumflex artery/ anterior interventricular artery /roof of the left atrium (average) in placebo and dapagliflozin group

SUVmax and SUVmean measured in the EAT surrounding the left circumflex artery (Cx) and the roof of the left atrium (RLA) were significantly decreased in the dapagliflozin group compared to the placebo group (p = 0.01 SUVmean for Cx and RLA, p = 0.003 and p = 0.019 SUVmax for Cx and RLA in dapagliflozin group) Fig. 2 A–B. A numerical decrease in SUVmax and SUVmean was observed in the EAT surrounding the anterior interventricular artery and right coronary artery only in the dapagliflozin group (p = 0.09 SUV mean, p = 0.07 SUV max) Fig. 2 C–D. Non significant correlations were found between the reduction in EAT thickness and SUV and the improvement of coronary flow reserve in dapagliflozin group (data not shown).

**Fig. 3:**
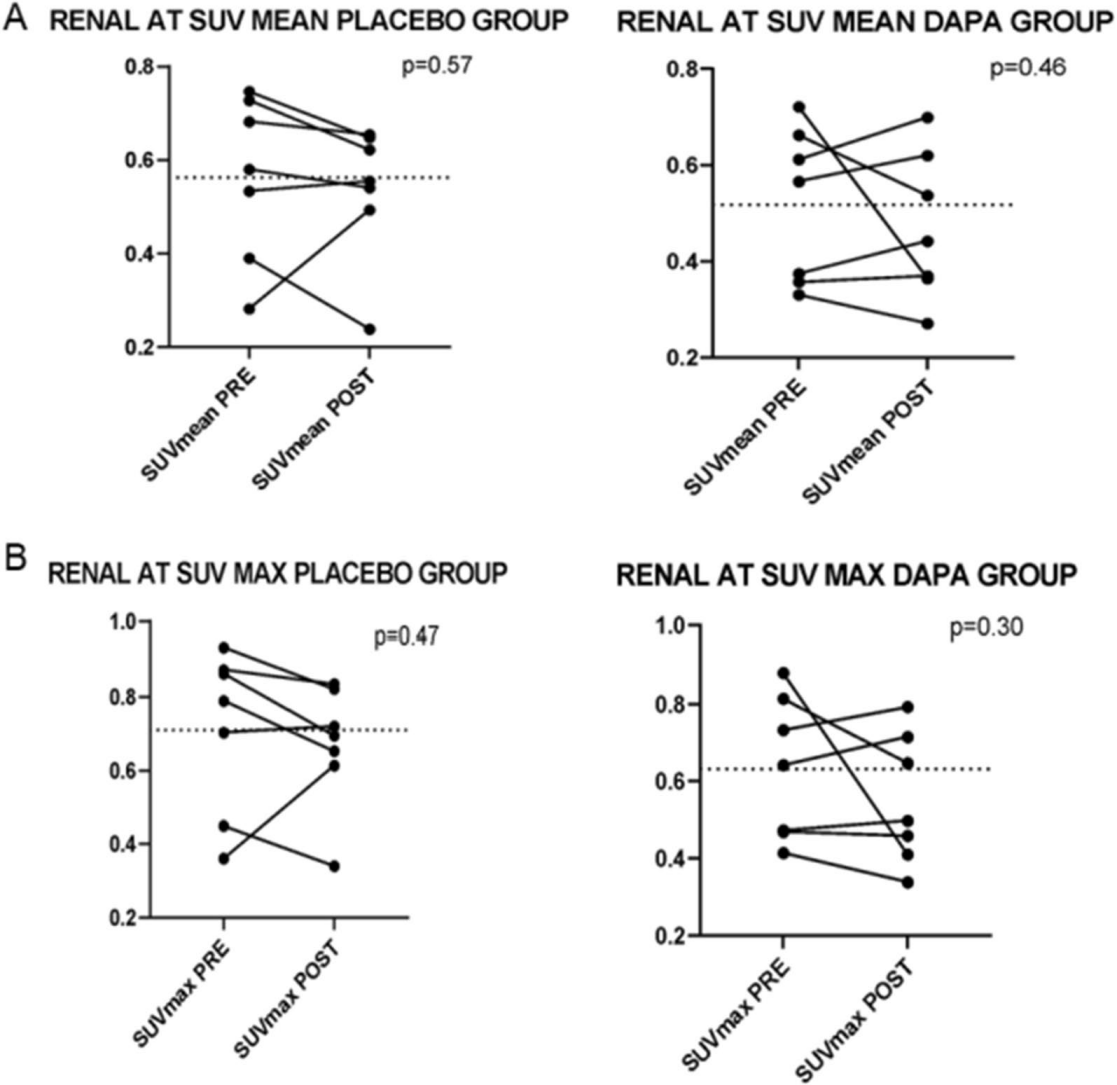
Perirenal adipose tissue metabolism (Renal AT): SUV mean (**A**) and SUV max (**B**) of perirenal adipose tissue in placebo and dapagliflozin group

**Fig. 4:**
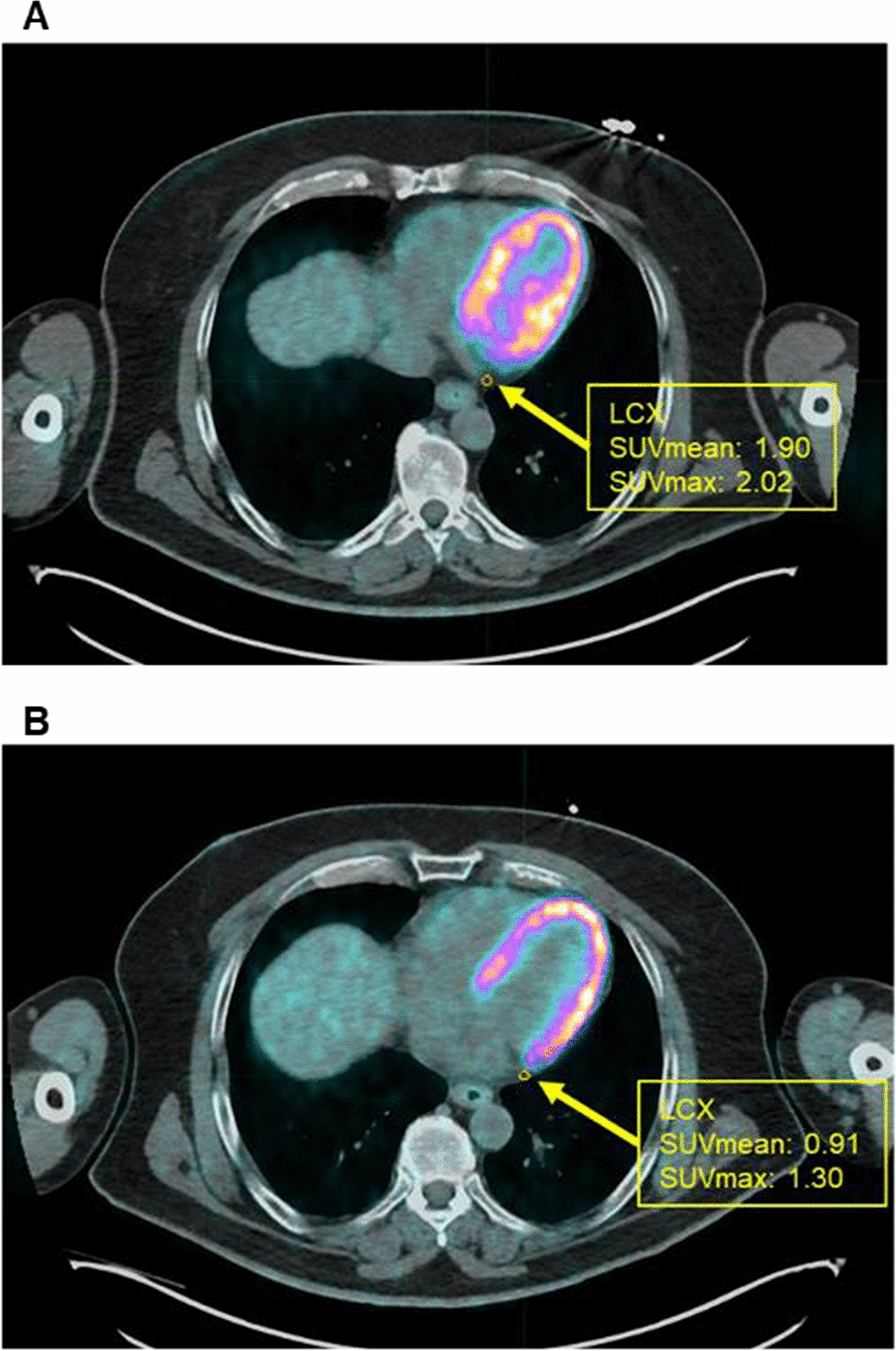
Epicardial adipose tissue FDG uptake: Representative FDG PET/CT scan of a patient of dapagliflozin group showing SUV mean and SUV max at left circumflex artery (CX) at baseline (**A**) and after 4 weeks of treatment (**B**)

No differences were found in perirenal adipose tissue SUV (Fig. 3 A–B) or in the other adipose tissue depots explored (mediastinal and subcutaneous) between the groups. In Fig. 4, A and B panels show SUV data obtained from a patient from the dapagliflozin group, at baseline (A) and after 4 weeks of treatment (B) respectively.

## Discussion

The beneficial effects of SGLT-2i in reducing the incidence of CV events and HF are well documented, but the mechanisms by which they exert these effects are still debated [15, 16].

The present study yielded important new findings: (1) the results demonstrate that 4 week treatment with dapagliflozin significantly reduced both thickness and FDG uptake in EAT (19% and 21.6% respectively); (2) this is the first study to demonstrate a selective effect of SGLT-2i on EAT compared to other visceral and subcutaneous adipose tissue depots; (3) the 21.6% reduction in EAT FDG uptake parallels the 30% improvement in myocardial flow reserve previously described [2], suggesting a link between these phenomena. Our results also demonstrate that the decrease in EAT is not correlated with changes in myocardial insulin sensitivity evaluated using the gold standard euglycemic insulin clamp method.

In line with previous studies, we saw a significant 19% reduction in EAT thickness [11, 17, 18] but we demonstrated for the first time that the effects of dapagliflozin occur after a relatively short treatment (4 weeks vs the previously described 24 weeks [17, 19–22]) and we demonstrated a selective effect on EAT compared to the other adipose tissue depots explored (visceral and subcutaneous).

The EAT is the most metabolically active adipose tissue [23] and this could be the reason why we observed a first selective response in this depot but, we cannot exclude that dapagliflozin treatment longer than 4 weeks could yield the same results in other visceral and subcutaneous depots.

Moreover, we are the first to find a 21.6% reduction in EAT SUV, which parallels the 30% improvement in myocardial flow reserve previously described [2]. In line with Katrine M. Lauritsen et al. [24], we did not observe any significant change in the other adipose tissue depots. In contrast, Esther Díaz-Rodríguez et al. observed an in vitro increase in EAT glucose uptake, but in samples obtained from heart surgery [25].

Adipose tissue and its proinflammatory state are known risk factors for CV diseases in T2D. In particular, inflammation of EAT, which anatomically is in direct contact with the myocardium [26], promotes oxidative stress and impairs endothelial and cardiac functions [7, 27–29]. Its peculiar anatomical characteristic, compared to all the other adipose tissue depots (i.e., the absence of a muscle fascia between EAT and the myocardium) means the two tissues are contiguous allowing crosstalk between EAT and the myocardium [10, 26]. It has a protective physiological function as it provides free fatty acids, secretes anti-inflammatory and anti-atherogenic adipokines, and provides a direct source of heat to the myocardium [30–33]. These positive effects are partly due to its origin from brown adipose tissue [34]. Several conditions like obesity and diabetes are associated with a reduction of beneficial brown fat activities [35, 36] and characterized by a decrease in brown adipocytes in favor of more unilocular white adipocytes. This leads to an increase of EAT volume and consequently of its pro-inflammatory activities [37].

We recently demonstrated that a 4 week treatment with the SGLT-2i dapagliflozin can significantly reduce resting myocardial blood flow leading to an increase in myocardial flow reserve in patients with T2D and stable CAD [2]. Myocardial flow reserve is the increase in blood flow through the coronary arteries above the normal resting volume and its reduction has been associated with a higher incidence of all-cause mortality [38]. The increase in myocardial flow reserve may be due to an improvement in the coronary microvascular dysfunction, which is a common characteristic of patients with type 2 diabetes [27, 39].

Since EAT and the myocardium share the same microcirculation [26], due to the abovementioned anatomical characteristics, we speculate that the reduction in EAT thickness reduces the imbalance between anti- inflammatory and pro- inflammatory EAT adipokine secretion with a significant amelioration of microvascular dysfunction.

Further, the interpretation of these results should consider that FDG PET/CT scan was performed during a hyperinsulinemic euglycemic clamp. Blood supply (i.e., myocardial blood flow in our study) has been described as an important factor for the regulation of in vivo insulin-mediated glucose uptake in both subcutaneous and visceral fat [40]. Thus, both insulin stimulation of adipocytes and the occurrence of inflammatory cells, like macrophages [41], may account for the observed metabolic change in adipose tissue. Indeed, reductions in the amount of adipose tissue have been widely associated with reductions in adipose tissue inflammation [42, 43], also recently confirmed in EAT [37], and it is well known that decreased inflammation determines a reduction in FDG uptake [44]. Thus, the EAT SUV reduction observed in the dapagliflozin group can be ascribed to a reduced macrophage activity. Unfortunately, probably as a consequence of the small sample size, we did not observe any significant correlation between changes in EAT thickness, SUV and myocardial flow reserve, which might have helped reinforce the interpretation of our data.

We can speculate that SGLT-2i treatment improves the anti-inflammatory role of EAT, alleviating microvascular dysfunction, which may explain the improvement in myocardial flow reserve described in our previous report [2] and the benefits of using SGLT-2i to reduce CV risk.

### Limitations and strengths of the study

This study has limitations. The number of subjects recruited was relatively small, which probably explains why some results trended toward, but did not reach statistical significance, limiting our conclusions to speculation. However, the reduction in EAT thickness and in EAT FDG SUV are statistically significant, and an increased study sample size would only have reinforced the significance**.** Moreover, an interim analysis was performed at enrolment of 30% of the patients (as set out in the protocol) [12]. Since the main secondary outcome of interest was statistically significant, we halted enrolment accordingly. In the protocol, we did not specify “EAT thickness” in the endpoints and, for this reason, the current analysis should be considered as a post-hoc sub-analysis.

Further, there is no measurement of local inflammatory markers, which could have supported our interpretation of the potential mechanism explaining the correlation between the reduction of EAT glucose uptake and reduction of myocardial blood flow. Lastly, localization of FDG uptake is difficult due to the small size of the coronary arteries, coronary motion, as well as increased FDG uptake in the myocardium during the hyperinsulinemic euglycemic clamp. Therefore, correction of cardiac and respiratory motion using dual-gated PET/CTA cardiac imaging may improve the detection of EAT FDG uptake [45].

On the other hand, our study also has many strengths. The use of a sophisticated and gold standard technique to synergically assess the morphology and metabolism of adipose tissue depots and myocardial blood flow allowed us to provide a pathophysiologic explanation for the beneficial effects of SGLT-2i treatment for CV diseases. Importantly, this is the first study to explore visceral and subcutaneous adipose tissue depots in the same subjects in response to SGLT-2i treatment. This allowed us to demonstrate the selective effect of a 4-week SGLT-2i treatment on EAT, explaining the rapid efficacy of these drugs in providing protection from CV events.

## Conclusions

The present study provides evidence that treatment with SGLT-2i dapagliflozin significantly reduces EAT thickness and EAT glucose uptake in patients with T2D and stable CAD. The decrease in glucose extraction parallels the decrease in resting myocardial blood flow, and the improvement in myocardial flow reserve. We speculate that SGLT-2i treatment restores the anti-inflammatory properties of EAT, which improves coronary microvascular dysfunction, thus providing a potential explanation for the previously described improvement in myocardial flow reserve [2]. These results help to focus the treatment of coronary microvascular dysfunction on specific pathophysiology rather than risk factors, leading to a precision medicine approach.

### Supplementary Information

**Additional file 1: Figure S1.** Trial design. Patients were randomized 1:1 to 4 weeks of dapagliflozin or placebo. At baseline and at the end of the intervention period, participants underwent FDG-PET/CT examination during euglycemic hyperinsulinemic clamp to assess morphometry and metabolic activity of visceral (epicardial, perirenal, mediastinal) and subcutaneous adipose tissue.

**Additional file 2: Figure S2.** Flowchart of study participants. Original with permission from L. Leccisotti et al. 2022, https://doi.org/10.1186/s12933-022-01607-4

## Data Availability

The datasets generated and/or analyzed during the current study are available from the corresponding author on reasonable request.

## Abbreviations

CAD: Coronary artery disease
EAT: Epicardial adipose tissue
HF: Heart failure
MGU: Myocardial glucose uptake
PET: Positron emission tomography
SGLT-2i: Sodium-glucose cotransporter-2 inhibitors
T2D: Type 2 diabetes
