# Supplementary figures and images for: Dapagliflozin treatment is associated with a reduction of epicardial adipose tissue thickness and epicardial glucose uptake in human type 2 diabetes
Source: Cardiovasc Diabetol. 2023 Dec 19;22:349. doi: 10.1186/s12933-023-02091-0 (PMC10731727; doi:10.1186/s12933-023-02091-0)

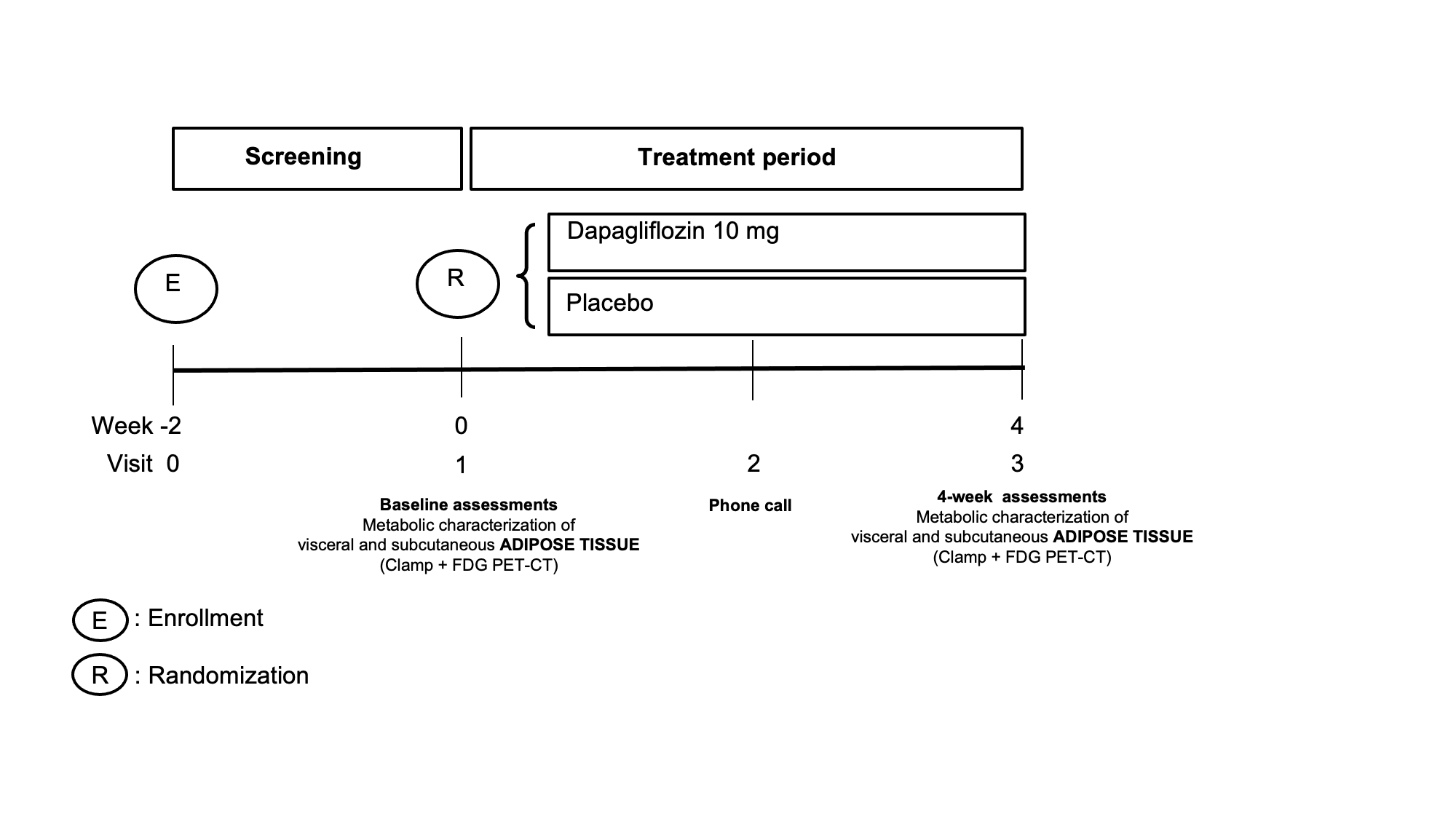

Supplement: Supplementary file 1 — Additional file 1: Figure S1. Trial design. Patients were randomized 1:1 to 4 weeks of dapagliflozin or placebo. At baseline and at the end of the intervention period, participants underwent FDG-PET/CT examination during euglycemic hyperinsulinemic clamp to assess morphometry and metabolic activity of visceral (epicardial, perirenal, mediastinal) and subcutaneous adipose tissue. [file 12933_2023_2091_MOESM1_ESM.tiff]

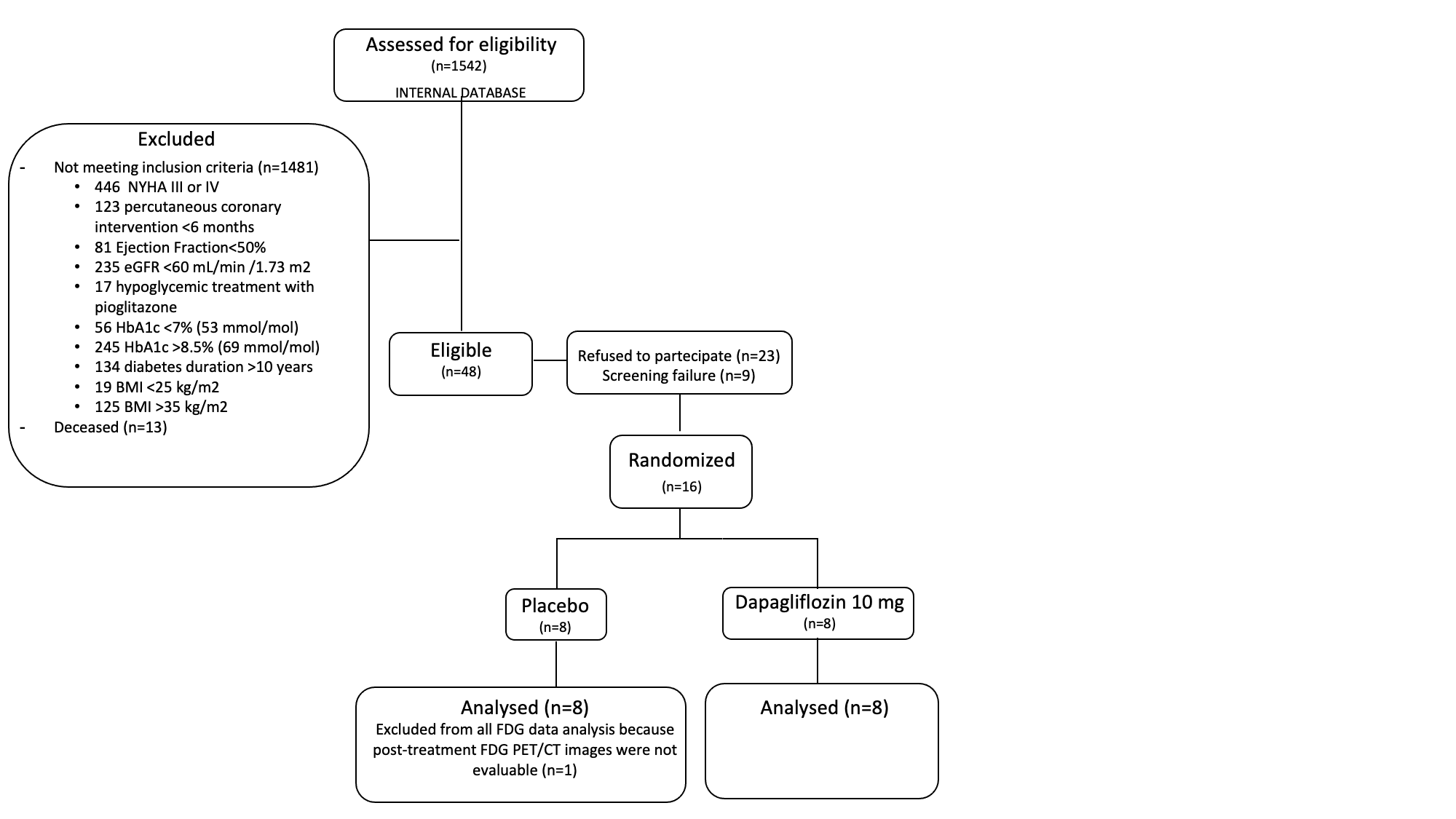

Supplement: Supplementary file 2 — Additional file 2: Figure S2. Flowchart of study participants. Original with permission from L. Leccisotti et al. 2022, https://doi.org/10.1186/s12933-022-01607-4 [file 12933_2023_2091_MOESM2_ESM.tiff]
